# Numerical investigation of crack propagation regimes in snow fracture experiments

**DOI:** 10.1007/s10035-024-01423-5

**Published:** 2024-04-22

**Authors:** Grégoire Bobillier, Bastian Bergfeld, Jürg Dual, Johan Gaume, Alec van Herwijnen, Jürg Schweizer

**Affiliations:** 1 WSL Institute for Snow and Avalanche Research SLF, Davos, Switzerland; 2 Institute for Mechanical Systems, ETH, Zurich, Switzerland; 3 Institute for Geotechnical Engineering, ETH, Zurich, Switzerland; 4 Climate Change, Extremes, and Natural Hazards in Alpine Regions Research Center CERC, Davos, Switzerland

**Keywords:** Snow Avalanche, Discrete Element Method Modeling, Crack Propagation, Supershear

## Abstract

A snow slab avalanche releases after failure initiation and crack propagation in a highly porous weak snow layer buried below a cohesive slab. While our knowledge of crack propagation during avalanche formation has greatly improved over the last decades, it still remains unclear how snow mechanical properties affect the dynamics of crack propagation. This is partly due to a lack of non-invasive measurement methods to investigate the micro-mechanical aspects of the process. Using a DEM model, we therefore analyzed the influence of snow cover properties on the dynamics of crack propagation in weak snowpack layers. By focusing on the steady-state crack speed, our results showed two distinct fracture process regimes that depend on slope angle, leading to very different crack propagation speeds. For long experiments on level terrain, weak layer fracture is mainly driven by compressive stresses. Steady-state crack speed mainly depends on slab and weak layer elastic moduli as well as weak layer strength. We suggest a semi-empirical model to predict crack speed, which can be up to 0.6 times the slab shear wave speed. For long experiments on steep slopes, a supershear regime appeared, where the crack propagation speed reached approximately 1.6 times the slab shear wave speed. A detailed micro-mechanical analysis of stresses revealed a fracture principally driven by shear. Overall, our findings provide new insight into the micro-mechanics of dynamic crack propagation in snow, and how these are linked to snow cover properties.

**Graphical Abstract:**

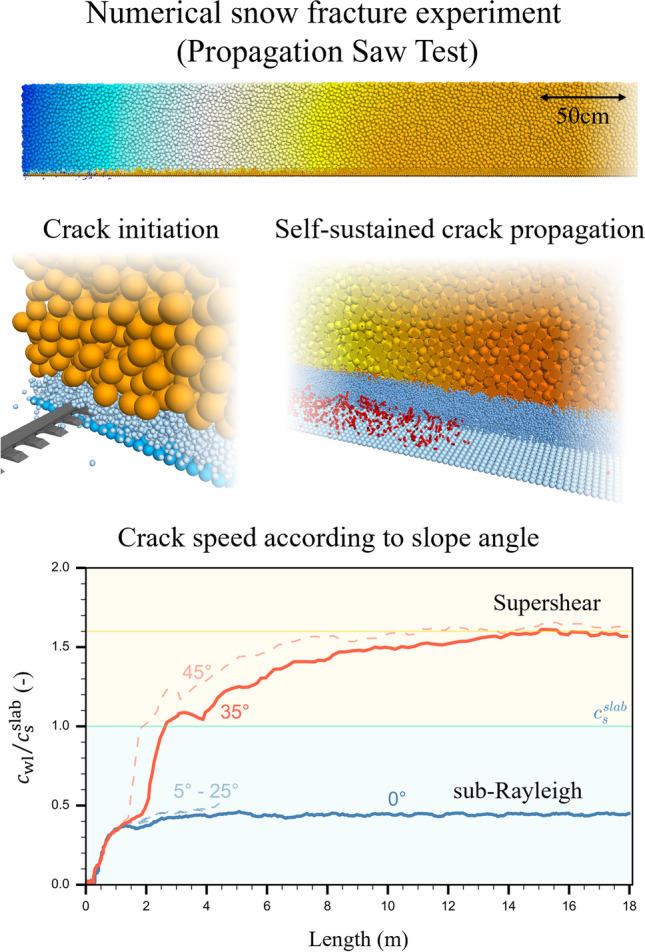

**Supplementary Information:**

The online version contains supplementary material available at 10.1007/s10035-024-01423-5.

## Introduction

When mountainous regions are covered by snow, a prominent natural hazard emerges with substantial destructive power: dry-snow slab avalanches. Forecasting the avalanche danger is of vital importance and relies on a solid understanding of avalanche release processes. Dry-snow slab avalanche release requires metastable snowpack conditions, where a highly porous anisotropic weak snow layer is buried below cohesive snow slab layers. A small perturbation, such as an additional load induced by a skier, may then lead to the formation of a crack which may propagate within the weak layer across the slope. Due to the highly porous nature of weak layers [e.g. 1], failure is accompanied by structural collapse, leading to vertical displacement of the slab [2–4]. This displacement leads to slab bending, which contributes to sustainable crack conditions at the crack tip even on flat or low-angle terrain [5]. During the propagation process, the slab may eventually fracture and detach if the slope angle is greater than the friction angle of the weak layer [approx. 30°; [6–8]. It is worth noting that crack propagation with the closure of crack faces on flat or low-angle terrain is often referred to as anticrack (or mode -I) [9]. This mode is known to occur under extremely large pressures (e.g. in deep earthquakes) or for highly porous materials (e.g. snow or firn); For snow, this has been extensively studied over the last two decades [2, 3, 10, 11].

During the last two decades, our understanding of slab avalanche formation processes has greatly improved, in particular through the development of a fracture mechanical field test: the Propagation Saw Test (PST) [2, 12, 13]. The PST consists of an isolating snow column containing a pre-identified weak layer. A crack is manually initiated in the weak layer with a snow saw until a critical crack length is reached, after which crack propagation is self-sustained without additional loading. Over the past years, the PST has been used to better understand the mechanisms driving the onset and dynamics of crack propagation [14]. Using a high-resolution Digital Image Correlation technique, Bergfeld et al. [3] related crack propagation speed obtained in a PST to slope-scale measurements and estimates obtained from an avalanche movie. In addition, several analytical and numerical models based on fracture and/or continuum mechanics were developed to investigate crack propagation and avalanche release [11, 15–21]. These models highlighted important aspects of the complex weak layer–slab interaction during crack propagation, in particular related to the stress states, as these cannot directly be measured.

While most numerical models are based on complex macroscopic constitutive laws, the Discrete Element Method (DEM) does not require such assumptions, as it is based on simple inter-particle contact models. From failure behavior in snow microstructure to large-scale crack propagation, DEM has shown its ability to simulate and analyze mechanical processes [21–25]. Originally in 2D, DEM simulations of PST experiments yielded good agreement with experimental data, yet a detailed analysis of the internal stresses during crack propagation was not possible [18, 21]. Recently, a 3D model was developed that accurately reproduced displacement fields, accelerations and crack propagation speed as observed in a PST experiment, allowing to investigate micro-mechanical processes [10]. Bobillier et al. [10] highlighted the emergence of a steady-state crack propagation regime after several meters of crack propagation. Yet, the mechanical properties influencing steady-state dynamic crack propagation still remain mostly unknown. While crack speed is expected to be bounded by the corresponding solid wave speed, Trottet et al. [26] recently suggested a transition to a supershear regime for crack speeds on avalanche slopes, in line with some indirect observations [27].

Our aim is to identify the mechanical drivers of the steady-state crack propagation regime in snow. To this end, we use a 3D DEM model of a PST to perform a sensitivity analysis mainly focusing on crack propagation speed. We first present a multidimensional parameter analysis on flat terrain emphasizing the influence of weak layer strength and elasticity. A semi-empirical model fitted to our simulations allows to better estimate crack speed at the slope scale. Simulations performed for different slope angles confirmed the existence of two steady-state crack propagation regimes with speeds either below or above the shear wave velocity of the slab layer; the latter was previously observed in earthquakes and termed supershear regime [28]. Our detailed analysis of slab displacements, stresses and failure states sheds light on the mechanisms involved in these different regimes.

## Methods

### Discrete element method and contact model

To generate a model of the propagation saw test (PST), we used the three-dimensional discrete element method (DEM). DEM, first introduced by Cundall, Strack [29], is a numerical tool including a large number of discrete interacting particles, commonly employed to study large deformations in granular-like assemblies. We used the PFC3D (v5) software (http://www.itascacg.com).

Here, we will only briefly introduce the DEM particle contact law. The parallel-bond contact model (PBM) we used was previously described in detail [10, 22]. The PBM component acts in parallel with a classical linear contact model and establishes an elastic interaction between the particles. Its mechanical parameters include the contact elastic modulus ${E}_{u}$, the Poisson's ratio ${\nu }_{u}$, the restitution coefficient ${e}_{u}$ and the friction coefficient ${\mu }_{u}.$ If particles are bonded, the bond part will act in parallel to the contact part. The bonded part is described by the bond elastic modulus ${E}_{b}$, the Poisson’s ratio ${\nu }_{b}$ and the shear and tensile strength ${\sigma }_{s}$ and ${\sigma }_{t}$. To reduce the number of variables we assume ${\nu }_{u}= {\nu }_{b}\triangleq {\nu }_{particle}$, ${E}_{u}= {E}_{b}\triangleq {E}^{particle}$ and ${\sigma }_{s}= {\sigma }_{t} \triangleq {\sigma }_{c}$. A linear relation between particle and macroscopic elastic modulus and between particle and macroscopic strength has been shown by Bobillier et al. [22]. Weak layer and slab macroscopic mechanical responses were studied with numerical load-controlled shear and compression tests, as presented by Bobillier et al. [22]. Reiweger et al. [30] showed that failure was almost independent of the loading rate in the brittle behavior range. Given the large strain rate during crack propagation (compressive strain rate: 2 s^−1^) [31], we assume here that sintering or rate-dependent effects would not affect the crack propagation behavior. Hence, our model does not include healing of broken bonds. The mechanical parameters between the weak layer and slab particles or the weak layer and base particles are the ones from the weak layer. The parameters are summarized in Table 1. Cundall-type numerical damping (${e}_{u}$) was used for the weak layer particles to prevent spurious oscillations affecting the stability of the system. However, no numerical damping was used for the slab particles because it modified the elastic wave speed in the slab which drive dynamic crack propagation [26]. The effect of numerical damping on simulation results is investigated in Appendices C and D, and discussed in Section. 4.

**Table 1:** Mechanical properties used in the DEM model. An asterisk (*) indicates those properties that were varied within the ranges indicated in square brackets

DEM mechanical property	
Poisson's ratio${\nu }_{particle}$	0.3
WL restitution coefficient${e}_{u}^{wl}$	0.1
Slab restitution coefficient${e}_{u}^{slab}$	1
Friction coefficient${\mu }_{u}$	0.5
Mean weak layer particle density (kg m^−3^)	550
* Mean slab layer particle density (kg m^−3^)	455, [363 – 545]
* Slab particle elastic modulus${E}_{slab}^{particle}$(MPa)	7, [1.35 – 33.6]
* Weak layer particle elastic modulus${E}_{wl}^{particle}$(MPa)	47.5, [14.25 – 109.25]
Slab bond strength (kPa)	infinite
* Weak layer bond strength${\sigma }_{c}^{wl}$(kPa)	144, [70 – 208]
PST mechanical property	
Mean weak layer density (kg m^−3^)	110
* Mean slab layer density (kg m^−3^)	250, [200 – 300]
* Slab elastic modulus${E}_{slab}$(MPa)	5.2, [1–25]
* Weak layer elastic modulus${E}_{wl}$(MPa)	1.0, [0.3 – 2.3]
* Weak layer tensile strength (kPa)	2, [0.98 – 2.9]
* Weak layer shear strength (kPa)	1.2, [0.59 – 1.73]
* Weak layer compressive strength${\sigma }_{{\text{wl}}}^{{\text{th}}}$(kPa)	4.3, [2.1 – 6.2]
System geometry	
Slab porosity	45%
Weak layer porosity	80%
Weak layer height (m)	0.02
Slab height (m)	0.4
* Slope angle$\psi$(°)	0 – 45

### DEM model of the propagation saw test

**Fig. 1:**
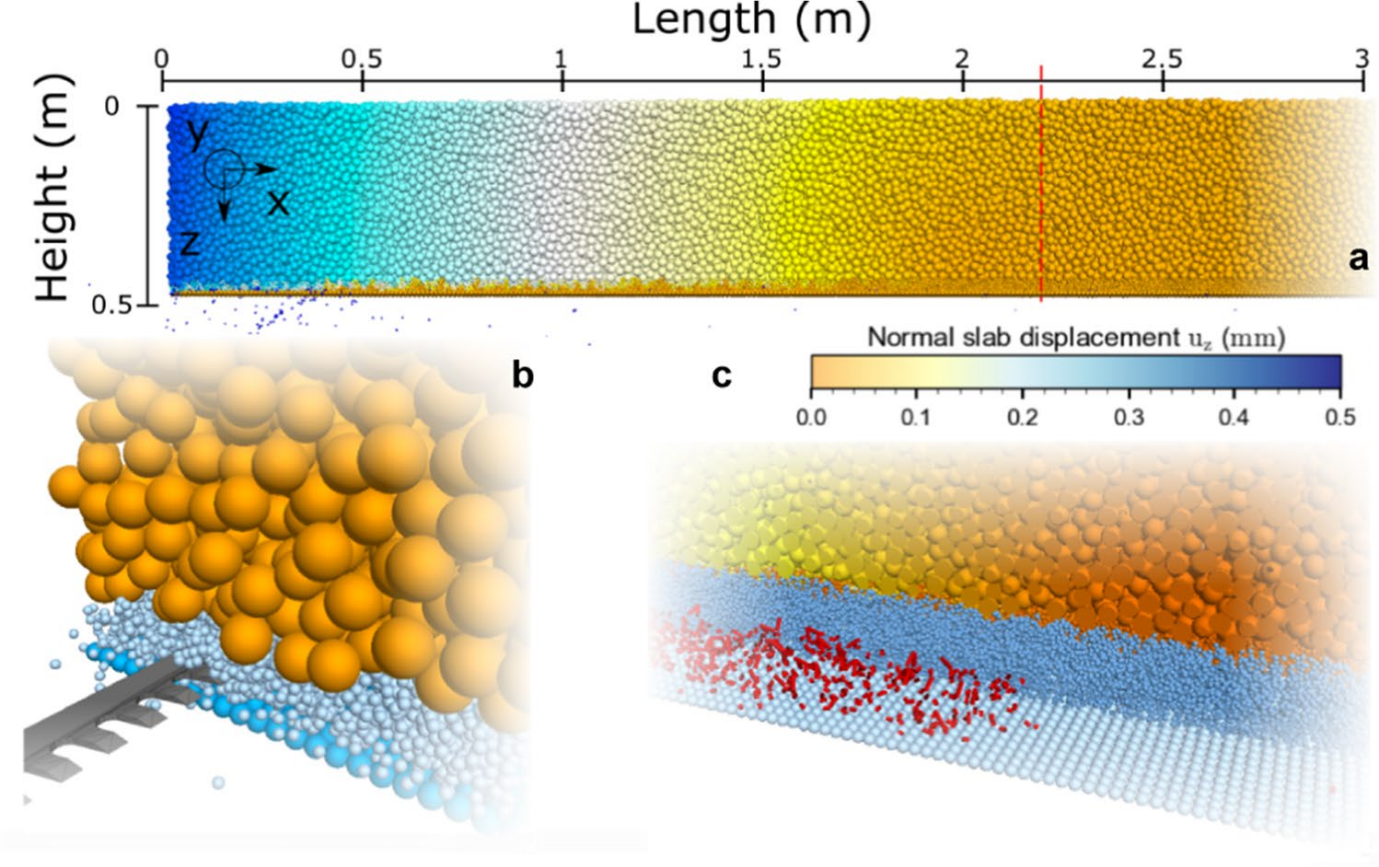
(**a**) Simulation of a PST experiment during self-sustained crack propagation (3 m of a 5 m long column is shown, crack tip at 2.2 m, red dashed line). Colored by its normal displacement (colored from no displacements in orange to 0.5 mm displacement in blue). (**b**) Simulation of PST during crack initiation phase, the snow saw is represented in dark grey. (**c**) Internal weak layer fracture process around the fracture process zone. Weak layer and slab were partly removed for visualizing broken bonds in the weak layer (in red; crack tip at 2.2 m)

The 3D DEM model of the PST experiment consisted of three layers: a rigid basal layer, a transversely isotropic weak layer, similar to layers of surface hoar or facets, and a dense and uniform slab layer (Fig. 1 and Supplementary movie). The basal layer was composed of a single particle layer (particle radius: *r* = 2.5 mm). The weak layer was created by cohesive ballistic deposition resulting in a porosity of 80% (particle radius *r* = 2.5 mm) with a layer thickness of 20 mm. Due to the deposition technique, the weak layer microstructure was unique. Applying homothetic transformation allows changing weak layer thickness while keeping its mechanical behavior [22]. The slab layer was generated by cohesionless ballistic deposition. The porosity of the slab layer was 45% (particle radius *r* = (10.5 ± 0.5) mm), slab thickness *H* = 0.4 m. The uniform variation in radius was introduced to prevent crystallization. At the particle scale (larger than the snow microstructure scale) the ice properties (e.g., strength, elastic modulus, Poisson’s ratio) cannot be used directly. Therefore, the particle scale can be considered as a mesoscale between the macroscopic scale (sample scale) and the microscale (individual snow grains made of ice). Hence, we adjusted the mesoscale particle density to obtain a realistic macroscopic snow density. For example, to set a macroscopic slab mean density of 250 kg m^−3^ given a slab porosity of 45% reached after ballistic deposition, the particle density is set as 455 kg m^−3^. It should be noted that the considered elements (mesoscale particles) have no true physical meaning and should only be regarded as entities of discretization similar to the mesh size in continuum models. As porosity can affect wave propagation, the wave speeds in the slab must be computed (see Section. 2.6).

The simulated PSTs were 5 m long and 30 cm wide; for analyzing the influence of slope angle on crack speed, the PST column length was extended to 20 m. Following guidelines for performing a PST in the field, the saw opened a crack while cutting the weak layer at a constant speed of 1 m s^−1^. The snow saw was modeled with a PFC3D rigid wall with dimensions similar to the saw used in field experiments (400 mm × 35 mm × 3 mm). The saw speed selected was twice as high as the experimental speed to reduce the simulation time but was still small compared to the crack propagation speed.

### Definition of crack tip and steady-state speed

**Fig. 2:**
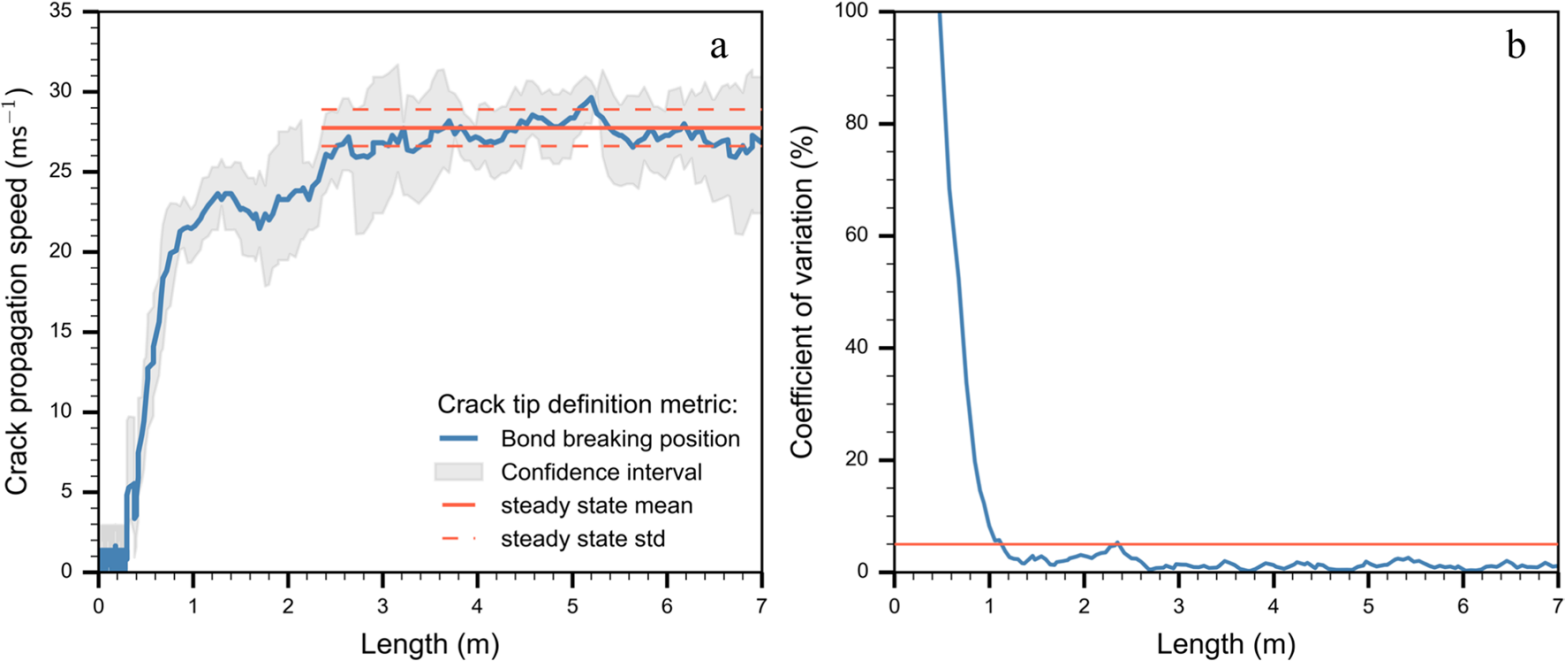
**a**) Crack speed (in blue) and confidence interval (grey envelope) along the simulated PST experiment; the orange solid line shows the mean (steady-state) crack speed between 2.35 and 7 m and the standard deviation (dashed lines). **b**) Coefficient of variation of crack speed along the column. The orange horizontal line corresponds to the threshold to define the steady-state (5%). PST properties: ${E}_{{\text{slab}}}=5.2 {\text{MPa}}$, ${E}_{{\text{wl}}}=1 {\text{MPa}}$, ${\sigma }_{wl}^{th}=4.3\mathrm{ kPa}$, $\psi =0^\circ$)

From the DEM simulations, we retrieved particle displacements, forces acting on particles and the positions of broken bonds. The stresses (${\sigma }_{zz},{\tau }_{zx}$) were calculated from the sum of measured forces at the interface between the basal layer and the weak layer. Bobillier et al. [10] tested several simulation outputs such as slab normal acceleration, normal slab displacement, weak layer stress tip, and weak layer bond-breaking position to define the crack tip position. It was shown that the bond breaking position was the most accurate metric because results did not depend on post-processing discretization length (${\Delta }_{x}=$ 2 cm). Crack speed was defined using $c=\Delta d/\Delta t$ where $\Delta d$ is the difference in crack tip position between two time steps and $\Delta t$ is the time step. To increase the precision of the speed data without distorting the signal, we used the finite difference method to approximate the derivative of the crack tip position as function of time employing Savitzky-Golay filtering. Crack speed was computed and filtered with a 9-point linear Savitzky-Golay filter [32]. We assumed crack speed to attain a steady-state value when the coefficient of variation of crack speed did not exceed 5% for the preceding 50 cm (Fig. 2b). In the example shown in Fig. 2, steady-state crack propagation was attained at about 2.35 m. The free boundary condition at the beam end creates an edge effect influencing crack speed [31]. To avoid the influence of edge effects at the far end of the column, we disregarded the speed values for the last meter for calculating the steady-state average crack speed.

### Parametric analysis

To investigate the influence of different mechanical properties on the crack propagation speed, we varied the slab elastic modulus, weak layer elastic modulus, weak layer strength (${E}_{{\text{slab}}}$, ${E}_{{\text{wl}}}$, ${\sigma }_{{\text{wl}}}^{th}$), and slope angle ($\psi$). The parametric analysis involved three steps. (1) We performed flat field simulations where only one system parameter was modified while the other two parameters remained unchanged. The constant parameter values were the same as used in Bobillier et al. [10] (${E}_{{\text{slab}}}=5.2 {\text{MPa}}$, ${E}_{{\text{wl}}}=1 {\text{MPa}}$, ${\sigma }_{{\text{wl}}}^{{\text{th}}}=4.3\mathrm{ kPa}$). (2) We performed flat field simulations varying all three parameters simultaneously (${E}_{{\text{slab}}}$, ${E}_{{\text{wl}}}$, ${\sigma }_{{\text{wl}}}^{{\text{th}}}$; 80 simulations). (3) We performed simulations with varying slope angle (increments of 10 degrees between simulations; ${\Delta }_{\psi }=$ 10°) keeping all other parameters constant as in (1). The ranges in elastic modulus and shear strength for slab and weak layer were based on literature values [33–35].

### Crack propagation speed analytical model

Heierli [36] proposed an analytical model based on the assumption of a solitary collapse wave. This model suggested that the crack in the weak layer occurs in the form of a localized disturbance zone propagating as a collapse wave with constant speed ${c}_{h} = \sqrt[4]{\frac{g}{2\left|h\right|}\frac{D}{\rho H}}$. Here $D$ is the flexural rigidity $D = \frac{{E}_{slab}{H}^{3}}{12(1-{\upnu }^{2})}$, *g* the gravitational acceleration, *h* the collapse height, $\rho$ the mean slab density, *H* the slab thickness and $\nu$ the Poisson’s ratio.

### Slab shear wave speed

**Fig. 3:**
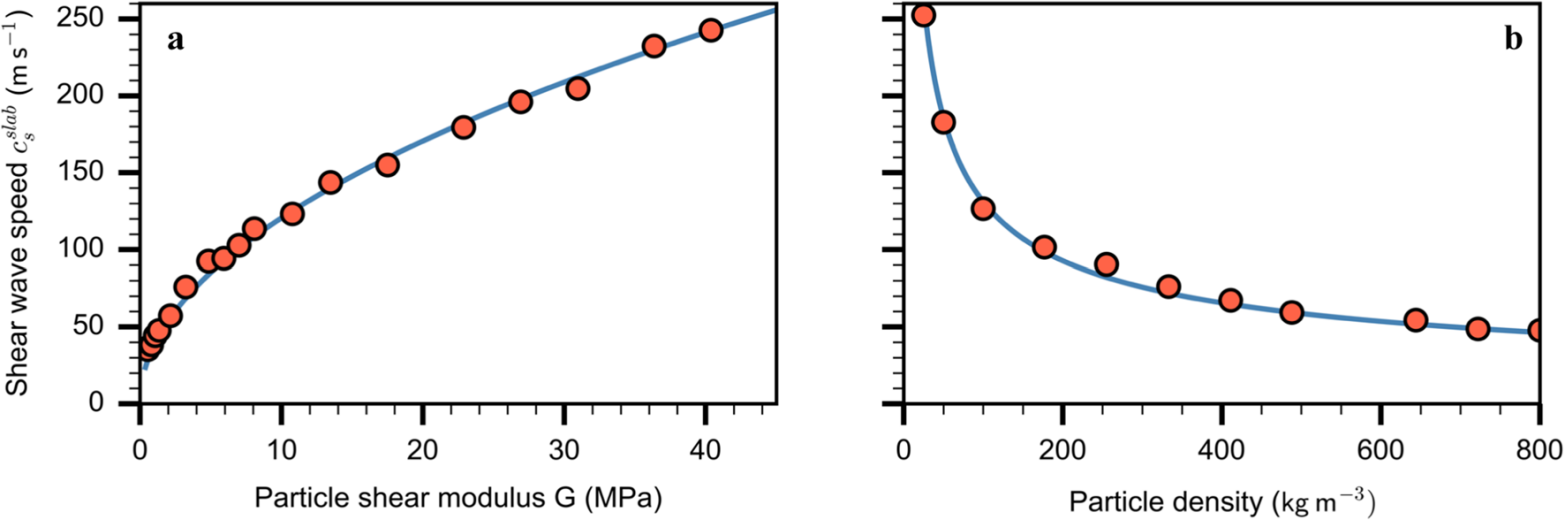
Slab shear wave speed (${c}_{{\text{s}}}^{slab}$) as a function of (**a**) particle shear modulus and (**b**) particle density. The orange dots show DEM simulation results and the blue line corresponds to the theoretical relation with an additional coefficient of 0.8 (Eq. 1) (*R*^2^ = 0.99)

As the surface wave speed should be the limiting speed for propagation of in-plane cracks [28], we determined the shear wave speed by applying a horizontal excitation to a thin band of particles at one end of the slab using a Heaviside step function. The system configuration consisted of the slab layer only, without gravity applied. The shear wave speed for the slab layer was then derived by measuring the travel time of the displacement wave along the column. Figure 3 shows the slab shear wave speed with particle shear modulus and density. The simulated wave speeds (orange dots) are in line with the theoretical relation. The fitting coefficient (0.8, *R*^2^ = 0.99) was introduced to account for the effect of the weak layer microstructure and depends on the fabric, particle size, and bond size (Eq. 1).

1 $${c}_{{\text{s}}}^{slab}=0.8\sqrt{\frac{{E}_{slab}^{particle}/2(1+{\upsilon }_{particle})}{{\rho }_{particle}}}$$

### Time step

The length of the time step was determined as function of the particle properties according to:

2 $$\Delta t \approx \, \text{f r}\sqrt{\frac{\rho }{E}}$$

where $\rho$ and $r$ are the smallest particle density and radius, respectively, $E$ is the largest bond or particle elastic modulus, and *f* is a safety factor (0.8). PFC3D software dynamically calculated the time step in this manner to ensure the stability of the DEM.

## Results

### Parametric analysis

#### Steady-state regime

During the propagation saw test, the crack propagates within the weak layer and reaches a steady-state speed, which depends on the mechanical properties of the system. To investigate the influence of different mechanical properties on the steady-state crack propagation speed in flat terrain ($\psi =0^\circ$), we varied the slab elastic modulus, weak layer elastic modulus and weak layer strength (${E}_{{\text{slab}}}$, ${E}_{{\text{wl}}}$, ${\sigma }_{{\text{wl}}}^{th}$).

**Fig. 4:**
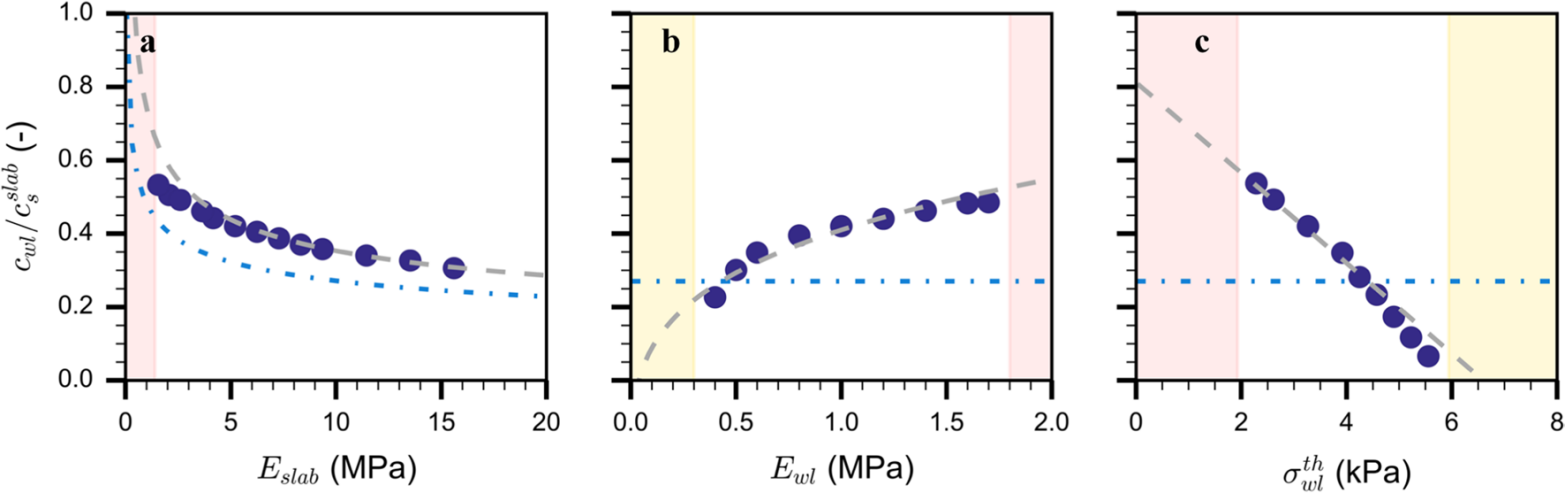
Normalized steady-state crack speed as a function of (**a**) slab elastic modulus, (**b**) weak layer elastic modulus and (**c**) weak layer strength. The dark blue dots correspond to the simulation results. The grey dashed lines correspond to the semi-empirical model (Eq. 3) fitted on data of 110 simulations (*R*^2^ = 0.96). The blue dotted dashed lines correspond to the results obtained with the analytical model by Heierli [36]. The yellow background indicates snowpack conditions where crack propagation cannot occur; the red background shows snowpack conditions where the weak layer spontaneously fails without any initial crack. The steady-state crack speed is normalized by the slab shear wave speed (${c}_{wl}/ {c}_{s}^{slab}$)

Crack propagation speed increased with weak layer elastic modulus and decreased with weak layer strength (Fig. 4b, c). In addition, an increase in slab elastic modulus led to an increase in crack propagation speed. However, a decreasing trend is observed after normalization by the slab shear wave speed (Fig. 4a). Crack speed predictions by the model of Heierli [36] were lower compared to our simulations (Fig. 4a), but showed similar behavior. Simulation results show (Appendix 11) that the collapse height *h *is about 50% of the weak layer height independent of the slab elastic modulus. The influence of weak layer properties (Fig. 4b, c) on crack speed cannot be represented by the model of Heierli [36], as it only contains the slab elastic modulus. Based on our simulations, we suggest a semi-empirical extension to the analytical model of Heierli [36]. Its form, i.e. the additional additive terms, was anticipated based on the multidimensional simulation results that suggest that the mechanical parameters are not correlated.

3 $${c}_{wl}=\sqrt[4]{-\frac{g}{2h}\frac{D({E}_{slab})}{\rho H}}+\sqrt[4]{{{\beta }_{1} E}_{wl}}-{\beta }_{2} {\sigma }_{wl}^{th}+\varepsilon$$

(${\beta }_{1}= 4 648 444.7$, ${\beta }_{2}=7.9$,$\varepsilon =-14.8$, grey dashed line in Fig. 4). Overall, this model was in good agreement with results from 110 simulations (*R*^2^ = 0.96).

The parameter analysis also revealed the influence of slab and weak layer properties on crack propagation propensity. Extremely soft slabs or a rigid weak layer made the system unstable, i.e. the weak layer directly failed and collapsed without propagation (Fig. 4, red background). The system can also be too stable for crack propagation, when the weak layer strength is high or the modulus is low (Fig. 4b, c: yellow background).

#### Propagation speed

**Fig. 5:**
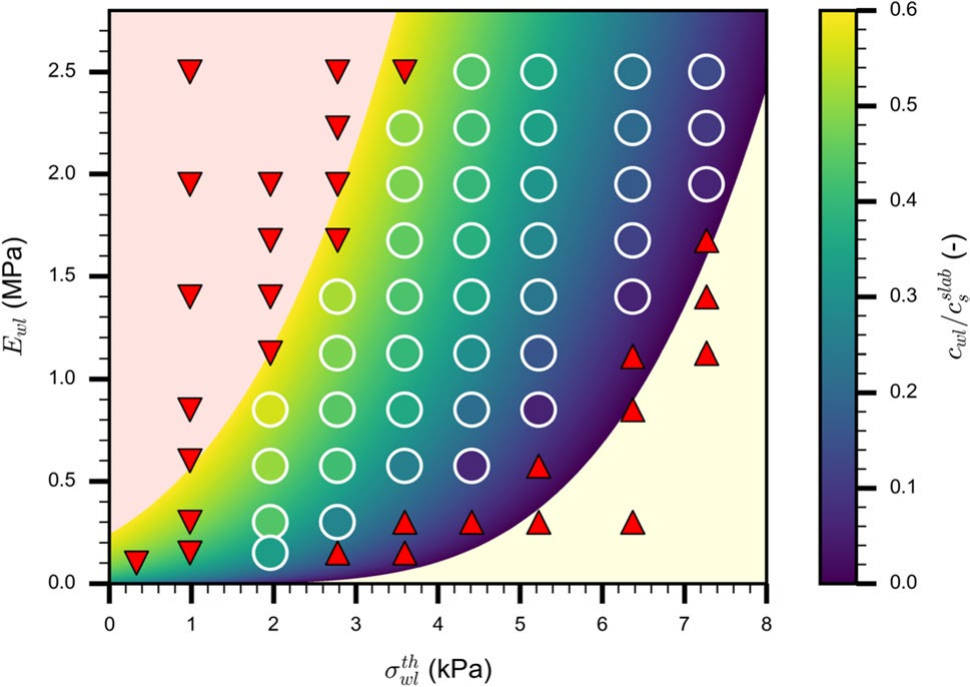
Normalized steady-state crack speed as a function of weak layer elastic modulus and weak layer strength (${E}_{slab}$ = 5.2 MPa). The colored dots (white outlines) represent individual simulations, the colored background corresponds to the semi-empirical model (Eq. 2). The yellow background indicates snowpack conditions where crack propagation cannot occur (up triangle for the simulations). The red background shows snowpack conditions where the weak layer spontaneously fails without any initial crack (down triangle for the simulations). The colormap is graduated with the crack propagation speed normalized by the slab shear wave speed (${c}_{wl}/ {c}_{s}^{slab}$)

Figure 5 shows how weak layer properties influence crack speed (colored dots) for the standard value of the slab elastic modulus (${E}_{{\text{slab}}}$ = 5.2 MPa; Tab. 1). The colored background corresponds to the semi-empirical model results and shows good agreement with simulation data. Depending on weak layer properties, we may have immediate failure of the weak layer (unstable system, red background in Fig. 5) or no propagation at all (stable system, yellow background in Fig. 5). Figure 5 highlights that crack propagation only occurred in a certain range of weak layer properties where the system was metastable. The crack propagates with a steady-state speed limited to 0.6 times the slab shear wave speed. In contrast, crack propagation propensity was not limited by slab elastic modulus, except for unrealistically soft slabs (red background in Fig. 4a). The findings shown in Fig. 5 also hold for stiffer slabs (${E}_{{\text{slab}}}$ = 15 MPa; *R*^2^ = 0.88; Appendix 10).

### Influence of slope angle

#### Crack propagation speed regimes

**Fig. 6:**
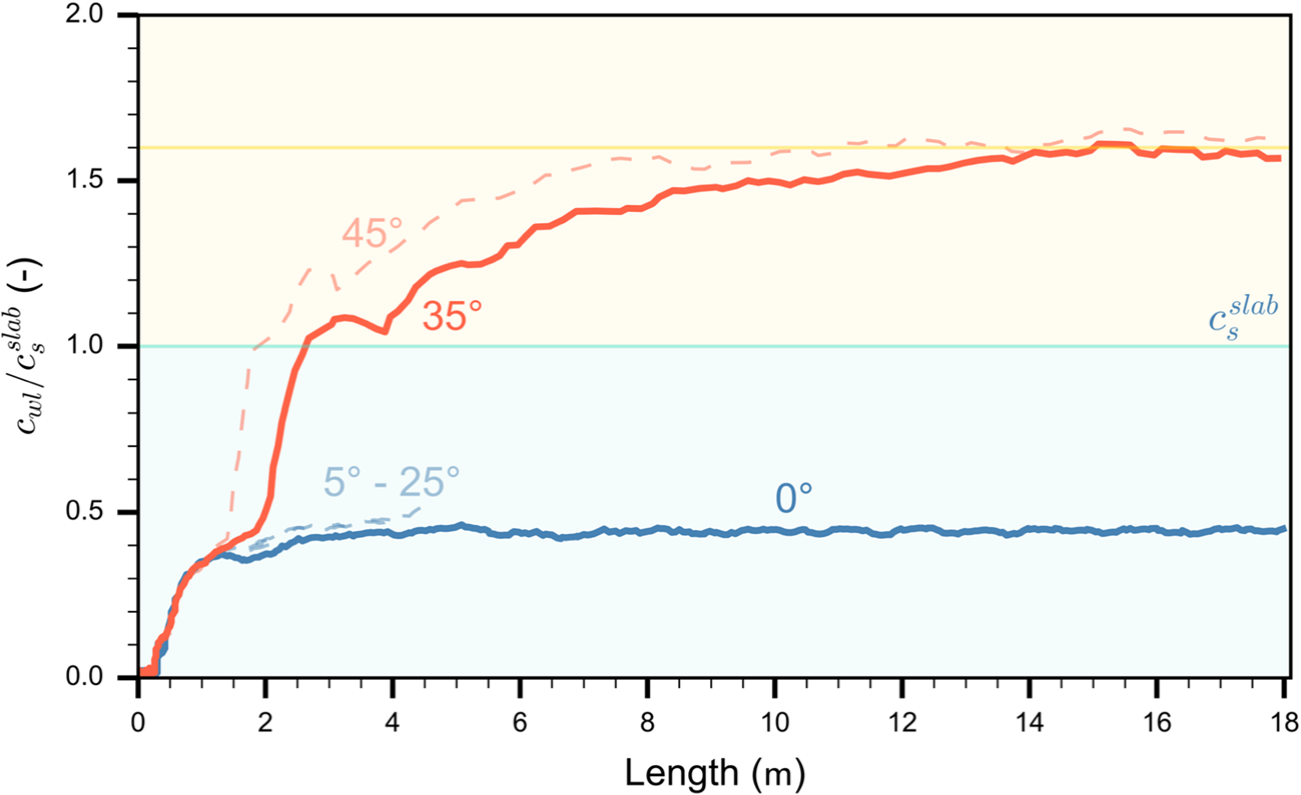
Normalized crack speed along the PST column for different slope angles ($\psi$). The blue line shows the crack speed evolution for a PST in flat terrain ($\psi$= 0°), the orange line that for a tilted PST ($\psi$= 35°). The dashed light blue lines correspond to the tilted PSTs with slope angles between 5 and 25°; the dashed light orange lines correspond to tilted PSTs with slope angle 45°. The blue background corresponds to the range of sub-Rayleigh crack speed (${c}_{wl}<{c}_{s}^{slab}$), the yellow background to the range of supershear crack speed (${c}_{{\text{wl}}}>{c}_{{\text{s}}}^{slab}$). The yellow line represents 1.6 ${c}_{{\text{s}}}^{slab}$

The slope angle of simulated PSTs (length: 20 m) was increased in steps of 10°, resulting in different crack propagation speeds. Overall, we observed two regimes (Fig. 6). From 0 to 25° incline, crack speed reached steady-state values after about 1.5 m and was independent of slope angle. Normalized steady-state crack speed was 40% of the shear wave velocity. From 30 to 45°, the crack speed evolution along the PST exhibited a more complex behavior. Focusing on the simulation results obtained with 35° slope angle (orange line in Fig. 6), the crack speed was very similar for the first 3 m to that on low angle terrain. Beyond 3 m, crack speed rapidly increased. The subsequent sharp increase in crack speed is mainly due to the crack tip definition we used, which is based on the bond breaking position. During the formation of a daughter crack ahead of the mother crack, the difference in crack tip position ($\Delta d$) between two time-steps strongly increased, inducing a jump in crack speed (Fig. 5). Beyond 5 m, crack speed continuously increased and after approximately 14 m it reached a relatively constant value of about 1.6 times the shear wave speed, suggesting a supershear steady-state regime. This behavior in crack speed was also observed for the other simulations with slope angle of 45°. Beyond 30°, slope angle mainly influenced the location where the transition from slow to rapid crack propagation occurred: the steeper the slope, the earlier the transition. The steady-state crack speed values on the steeper slopes were 1.6 times the shear wave speed. Therefore, we denote this type of propagation as supershear in analogy to supershear fracture speeds observed in other materials and earthquakes [e.g. 37, 38]. For slope angles larger than 45°, our system was unstable, since the weak layer immediately failed.

#### Slab displacements analysis

**Fig. 7:**
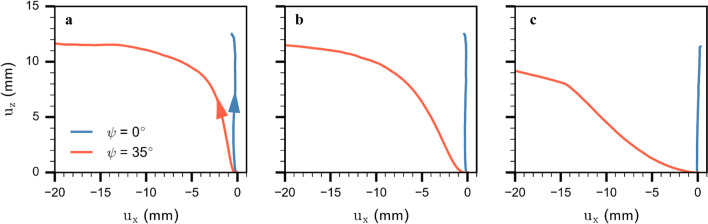
Vertical vs. horizontal displacement for two slope angles at three different positions along the PST: (**a**) at 1.5 m, (**b**) 3.2 m, and (c) 12 m. Blue lines show slab displacements at 0° and orange lines at 35° slope angle. Arrows in (**a**) indicate temporal evolution

We choose two slope angles (0° and 35°, blue and orange line in Fig. 6, respectively) for a more detailed analysis of the mechanical behavior during crack propagation. We analyzed slab displacement at three positions along the PST column (1.5 m, 3.2 m and 12 m). Slab displacement dynamics exhibited different behavior depending on slope angle. For the horizontal PST, the slab mainly moved downwards (${u}_{z}>0$, ${u}_{x}\approx 0$) regardless of the position in the column, indicating that weak layer structural collapse was the main driver of slab displacement (blue lines in Fig. 7). This suggests that the fracture mode in the weak layer was mainly due to compression (so-called mode I anticrack). For the tilted PST (35°), at 1.5 m, the slab first moved mainly downwards due to weak layer collapse (${u}_{z}>0$,${u}_{x}\approx 0$), before slab displacement transitioned to a sliding behavior ${(u}_{z}\approx$ constant). At 3.2 m, slab displacement showed a similar behavior, although the slope parallel displacement became more prominent more quickly (Fig. 7b). Finally, at 12 m, slab displacement started with slope parallel movement for approximately 2 mm (${u}_{z}\approx 0$,${u}_{x}<0$), before the normal displacement became apparent, resulting in a collapse and sliding behavior (Fig. 7c). The initial displacement thus suggests that the weak layers failed mainly in mode II (shear).

#### Bond breaking distribution and stress concentration

**Fig. 8:**
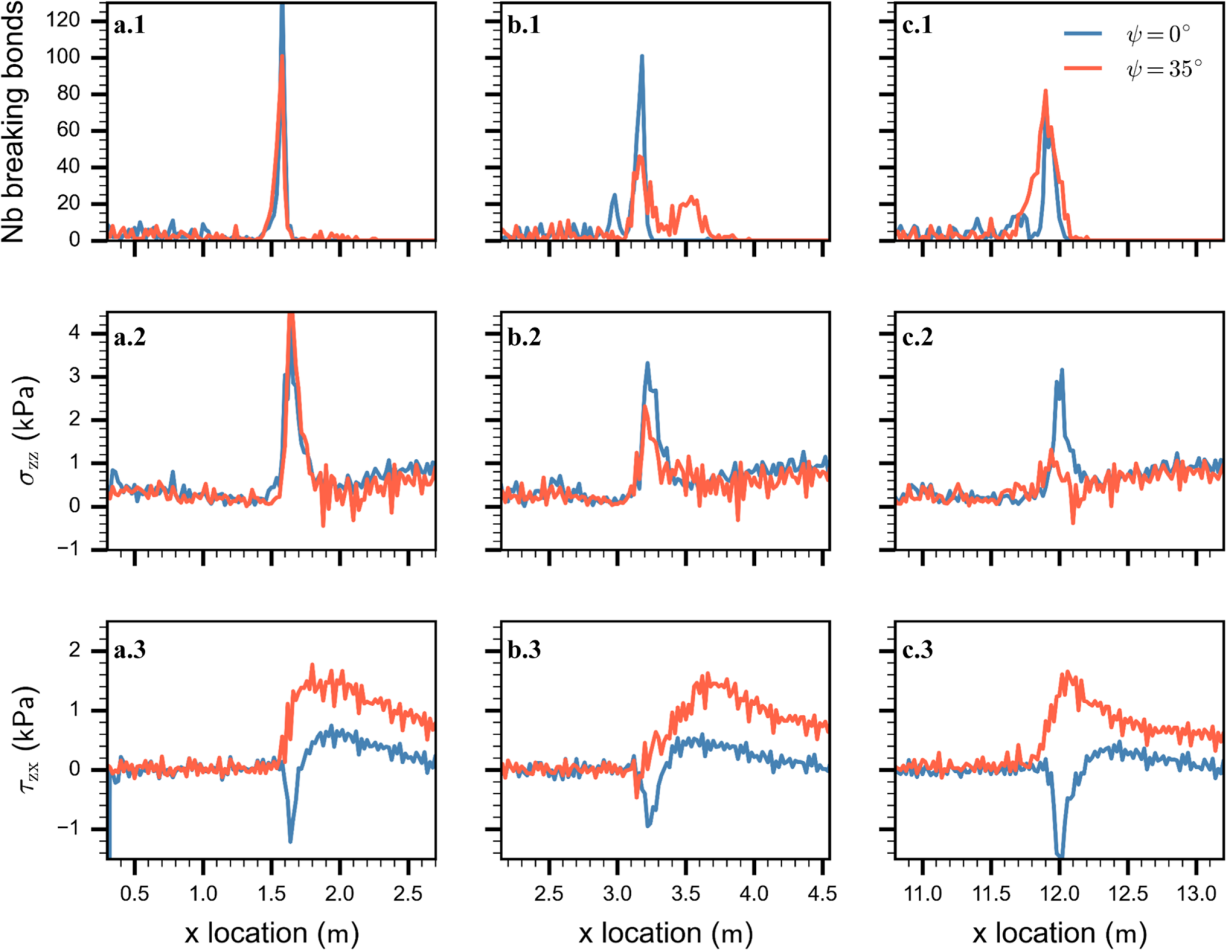
Micro-mechanical behavior during crack propagation for two slope angles. (**a**, **b**, **c**) correspond to different crack tip locations respectively: 1.5 m, 3.2 m, 12 m. (1) Weak layer bond breaking distribution along the column length. (2) Normal stress ${\sigma }_{{\text{zz}}}$ and (3) shear stress (${\tau }_{{\text{zx}}}$) along the length of the PST column. Blue lines for the level PST at 0° and orange lines for the inclined PST at 35°

**Fig. 9:**
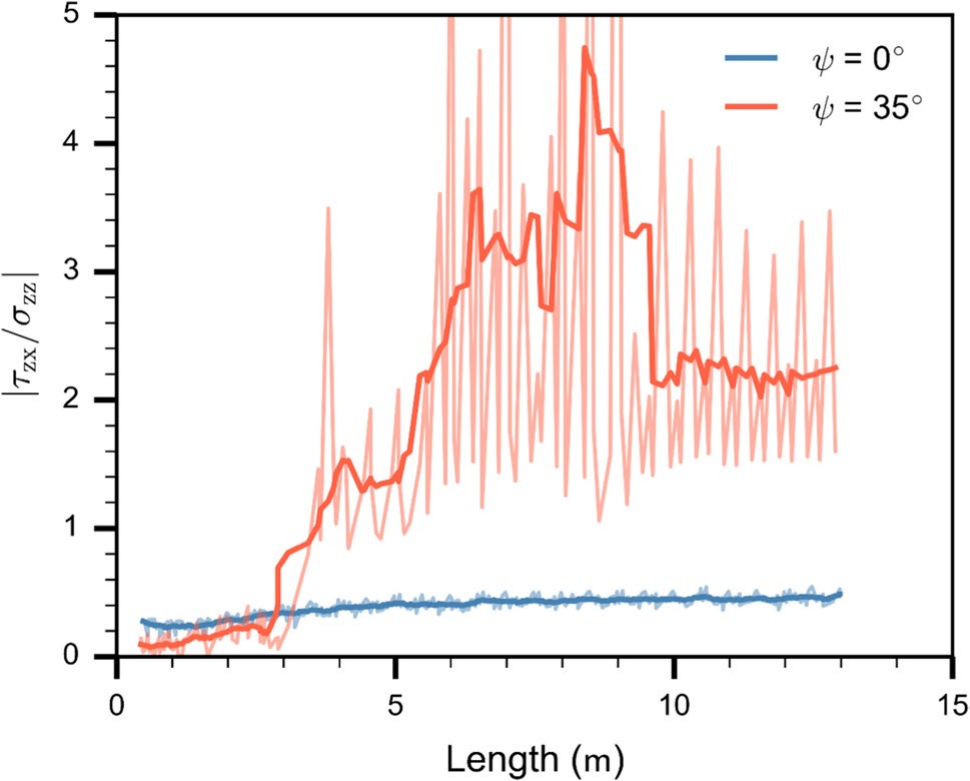
Absolute ratio between shear stress (${\tau }_{{\text{zx}}}$) and normal stress (${\sigma }_{{\text{zz}}}$) at the crack tip along the length of the PST column. Blue line corresponds to 0° and orange line to 35° slope angle. Light color lines show the raw data, which were smoothed using a Savitzky–Golay filter (dark color lines)

We investigated micro-mechanical quantities along the PST column: weak layer bond-breaking distribution, normal and shear stresses at the top of the substratum (Fig. 8). As expected, based on slab displacements, for the non-inclined PST ($\psi$= 0°), the micro-mechanical behavior did not significantly change along the PST column (blue lines in Fig. 8). Most of the weak layer bonds were breaking in a small area about 20 cm wide. In the steady-state regime, i.e. after 3 m, peak stresses indicated a mixed-mode failure with a main normal stress component (${{\tau }_{{\text{zx}}}\approx 0.45 \sigma }_{{\text{zz}}}$, Fig. 9).

For the tilted PST (35°), a clear regime transition appeared along the PST column, from a failure mainly driven in mode I (mixed-mode I/II) to mode II (mixed-mode II/I). Around 1.5 m, the bond breaking distribution and the normal stress are similar to those found for the horizontal PST. The shear stress is also similar yet larger due to the slab weight (Fig. 8). At about 3 m, where the crack speed strongly increased (orange line in Fig. 6), the failure mode started to change from normal stress driven to shear stress driven (orange line in Fig. 9). The weak layer bond breaking distribution showed two clusters at 3.2 m and at 3.6 m related to the peaks of normal and shear stress, respectively (Fig. 8b). This emergence of a secondary crack is similar to what is described as the Burridge–Andrews mechanism in supershear earthquakes, where a daughter crack forms ahead of the mother crack. At about 12 m, where the crack speed reached a supershear steady-state regime (Fig. 6), the bonds were breaking over a wider area compared to the PST in flat terrain. Stresses indicated a weak layer failure mainly driven by shear (${{\tau }_{{\text{zx}}}\approx 2 \sigma }_{{\text{zz}}}$, Fig. 9).

#### Shear to normal stress ratio at the stress tip

We tracked the ratio of shear to normal stress at the crack tip. On the flat, at the beginning of propagation, the ratio was low due to high normal stresses at the crack tip. The ratio somewhat increased up to about 4 m due to the addition of the upslope shear component induced by slab bending. After 4 m, the stress ratio reached a steady-state indicating a mixed-mode fracture dominated by the normal stress component (Fig. 9, blue line). For the tilted PST (35°), the stress ratio was initially low due to a large normal stress component while the shear component induced by slab bending was compensated by the downslope shear stress induced by gravity. From 3 to 10 m, where the crack speed diverged from the behavior in the horizontal (Fig. 6), the stress ratio at the crack tip was in a transition phase corresponding to increasing crack speed. From 10 to 13 m, the stress ratio reached a stable value suggesting steady-state crack propagation in mixed-mode driven by the shear stress component (Fig. 6 and 8, orange line). The light color lines show the raw data where the stress discretization induces the oscillations(Δx = 2 cm).

## Discussion

We used a 3D discrete element model of a PST experiment to investigate the effect of mechanical properties and slope angle on the crack propagation regime during snow fracture experiments. Microscopic model properties were adapted to observed macroscale snowpack properties as demonstrated by Bobillier et al. [22]. Crack speed was determined based on bond-breaking position [10]. We focused on steady-state crack propagation to study the influence of selected mechanical properties of slab and weak layer. While our study focuses on the dynamics of crack propagation, the simulated set-up allowed us to obtain realistic values of critical crack length, typically between 0.15 and 0.45 m, in line with experimental data [39]. Therefore, the presented simulation results could be used to study the onset of crack propagation. Results highlight the influence of weak layer properties on crack speed in low angle terrain. Specifically, crack speed decreased with increasing weak layer strength or slab elastic modulus and increased with increasing weak layer elastic modulus. On steep slopes, we observed a transition from sub-Rayleigh to supershear crack propagation and highlighted a change in fracture mode.

Since DEM does not require the assumption of a complex macroscopic constitutive model, it is considered a valuable tool for numerical studies of snow deformation and failure. Gaume et al. [21] suggested a 2D DEM model with a simplified snow layer structure to capture the main ingredients required for dynamic crack propagation. However, the model geometry was too simplistic to analyze the micro-mechanics. To overcome these limitations, Bobillier et al. [22] generated a 3D DEM PST model with a complex, layered microstructure. Bobillier et al. [10] then showed this model to accurately reproduce experimental crack propagation, including weak layer structural collapse, and to provide insight into the micro-mechanical failure processes. In the present study, we increased the simulation domain (up to 20 m long PST columns) and extended the micro-mechanical analysis to investigate the influence of various mechanical parameters on crack propagation.

On low angle terrain, PST field experiments are well suited to estimate crack propagation propensity up to the slope scale (10 m), as shown by the experimental results of Bergfeld et al. [3]. While field experiments are inherently more trustworthy, they cannot readily be performed as specific natural conditions are needed for crack propagation. In contrast, numerical experiments are more versatile and, for instance, suited to explore the many different combinations of parameters. This allowed us to show that crack propagation only occurred in a certain range of weak layer properties, whereas the slab modulus did not affect crack propensity.

Since the weak layer generation in our DEM model is complex [22], we cannot easily explore the influence of weak layer thickness. While Gaume et al. [18] suggested that weak layer thickness has no effect on propagation speed, the solitary wave model developed by Heierli [36] includes the weak layer collapse height – yet no other weak layer properties. To account for the influence of weak layer properties on crack speed, we therefore extended the formulation by Heierli [36]. While this would allow using our model with parameters from a manually observed or simulated snow profile, currently, no method exists to link weak layer collapse height to its microstructure. Therefore, in our semi-empirical model, the collapse height was assumed to be 50% of the weak layer thickness, based on simulation results. Implementing the measured collapsed height did not substantially improve the agreement between our semi-empirical model and the simulations (Appendix 11). The semi-empirical model offers a simple crack speed prediction and a promising functional form for further analytical model investigation. Gaume et al. [18] suggested a similar relation between mechanical parameters and crack speed on flat terrain. However, they presented results from numerical PST experiments with a length of 2 m, which suffer from edge effects. For titled PSTs, Gaume et al. [18] showed a linear influence of slope angle with only two distinct behaviors (below and above the snow friction angle). The difference with our results again seems to be related to the limited (too short) length of their PST model geometry.

Our results obtained for titled PSTs revealed two different crack propagation regimes: (1) sub-Raleigh crack propagation for slope angles < 30° and (2) supershear crack propagation for slope angles ≥ 30°. For regime (1), crack propagation dynamics were very similar and mostly independent of slope angle (Fig. 6). As these experiments were performed below the snow friction angle (approx. 30°), no additional shear force was applied after weak layer fracture. Stress concentrations at the crack tip remained similar throughout the PST and were dominated by the normal stress component (Figs. 7 and 8). In this range of slope angles, crack speed can be predicted by our semi-empirical model.

For regime (2), crack dynamics were completely different. Crack propagation speed reached a steady-state value larger than the slab shear wave speed. Our micro-mechanical analysis showed that in this regime fracture was mainly driven by shear stress (Figs. 7 and 8). Using a model based on the Material Point Method (MPM), Trottet et al. [26] recently showed a similar regime transition. Our results provide additional insight into the micro-mechanics and confirm the transition akin to the Burridge-Andrews mechanism (Fig. 8b). Indeed, we observed a daughter crack ahead of the mother crack induced by shear stress, as also reported by Trottet et al. [26]. Earthquake ruptures exhibit similar mechanisms during supershear rupture propagation [40–42]. In accordance with Trottet et al. [26], we showed that below the snow friction angle (regime 1) crack propagation is a rather complex mechanism involving the weak layer-slab interaction and that crack speed can be approximated with a semi-empirical model. Above the snow friction angle, in the supershear crack propagation regime, a pure shear model including the slab elastic modulus seems sufficient to approximate crack speed, as suggested by Trottet et al. [26].

While field experiments are limited to non-invasive data acquisition, DEM is a powerful tool to study internal processes for conditions with large deformation. We showed that the slab displacement field is a relevant indicator to estimate the propagation regime. In the supershear regime, slab movement started in slope-parallel direction, while in contrast, in the sub-Rayleigh regime, initial slab displacement was vertical. This observation would be a key indicator to confirm the supershear regime in field experiments, and could be derived with the digital image correlation technique as used by Bergfeld et al. [31].

While our model offers new insights into crack propagation dynamics, computational power is the main limiting factor for further investigation. The PST model with a length of 20 m requires (with a 2 ms data acquisition time) more than two weeks of computing time on a powerful workstation (Intel Xeon CPU 2.60 GHz, 14 Cores, RAM 256 Gb). Hence, mechanical parameter analysis during the supershear regime or larger or three-dimensional simulation domains are, unfortunately, not feasible with the present computational setup. For this purpose, we suggest using continuum approaches [e.g. 26, 43]. In addition, in our DEM model, the slab is purely elastic, therefore all deformation is recoverable, which allows the simulation to reach the supershear regime on steep slopes. Slab fractures would disrupt the supershear transition by changing the stress state. However, for hard slabs with higher strength, Trottet et al. [26] have shown the supershear transition is not affected.

Our DEM bond contact model is purely brittle and no energy is dissipated after bond breaking. Nevertheless, the weak layer microstructure introduces macroscopic dissipation similar to weak snow layer behavior. We applied numerical damping to reproduce metastable weak layers failing under expected loading conditions. Two types of damping exist: Cundall-type numerical damping (global damping [29]) and viscous contact damping. In principle, viscous contact damping would be preferred. However, it does not prevent the system from oscillating and becoming unstable under theoretically stable loading conditions. For this reason, we applied Cundall-type numerical damping, but only to particles of the weak layer. In all the simulations presented above, no numerical damping was used for the slab. As shown in Appendix 12 and 13, numerical damping in the slab affects the shear waves, and thus the crack propagation dynamics. However, it impacts neither the stress distribution nor the general conclusions regarding the supershear transition. Moreover, our DEM model does not consider particle compaction. Instead, Cundall-type numerical damping implicitly encompasses different sources of energy dissipation that are not explicitly modeled here (snow visco-plastic deformation, fast sintering). In the future, a user-defined bond damping model should be implemented (similar to the frequency-dependent damping, Rayleigh damping in classical FEM models).

Weak layer crack propagation can be divided into three regimes, the onset of crack propagation, the transitional regime, and the steady-state regime. Here we focused on the steady-state regime, linking crack speed to mechanical parameters. In the future, our numerical data could also be used to explore the transitional regime, and the onset of crack propagation, both key indicators to predict avalanche probability, location and size. In field experiments, the column length in PSTs is generally rather short, typically less than 2 m. Studying the transitional regime may help to link PST results obtained with short columns to the steady-state speed regime.

## Conclusions

Understanding crack propagation behavior in the highly porous and anisotropic material snow is crucial to appropriately model snow slab avalanche release. In this study, we evaluated the influence of different mechanical properties on steady-state crack propagation using a 3D DEM model. For the first time, we simulated large scale snow fracture experiments up to 20 m long, consisting of more than 930 000 DEM particles (1 800 000 contacts) to investigate the micro-mechanics of dynamical crack propagation. On the flat, model results highlighted the importance of the weak layer properties to initiate crack propagation and to estimate crack speed. Based on a multidimensional parametric study, we developed a semi-empirical model to predict crack speed from snowpack properties. Our results suggest a transition in the fracture mode at the snow friction angle. For slope angles larger than the snow friction angle, we report supershear crack propagation with crack speeds greater than the shear wave speed of the slab. In this regime, the fracture is mainly driven by shear, as demonstrated based on a detailed micro-mechanical analysis of stresses for weak layer failure. Our numerical findings indicate that the crack propagation speed measured in the flat during small-scale experiments may not necessarily be representative of up-slope/down-slope crack speeds in slope-scale crack propagation as involved in avalanche release.

In the future, we plan to study the transitional crack speed regime in more detail to gain a better understanding of dynamical crack propagation during short PST experiments. In addition, an in-depth analysis of the effect of slab fracture, topographic and three-dimensional effects should bring us closer to improving the prediction of avalanche release sizes from snowpack properties.

### Electronic supplementary material

Below is the link to the electronic supplementary material.

Supplementary file1 (MP4 36240 KB)

## Figures and Tables

**Fig. 10:**
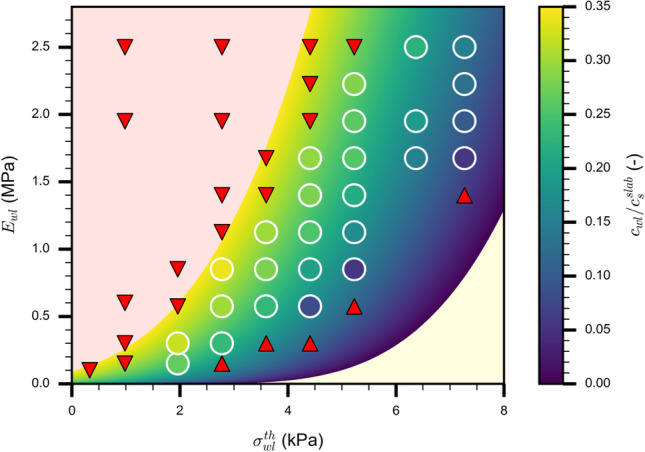
Normalized steady-state crack speed as a function of weak layer elastic modulus and weak layer strength, with a slab elastic modulus ${E}_{{\text{slab}}}$ = 15 MPa (same plot as in Fig. 4 where ${E}_{{\text{slab}}}$ = 5.2 MPa). The colored dots (white outlines) represent individual simulations, the colored background corresponds to the semi-empirical model (Eq. 2). The yellow background indicates snowpack conditions where crack propagation cannot occur (up triangle for the simulations). The red background shows snowpack conditions where the weak layer spontaneously fails without any initial crack (down triangle for the simulations). The colormap is graduated with normalized the crack propagation speed by the slab shear wave speed (${c}_{wl}/ {c}_{s}^{slab}$)

**Fig. 11:**
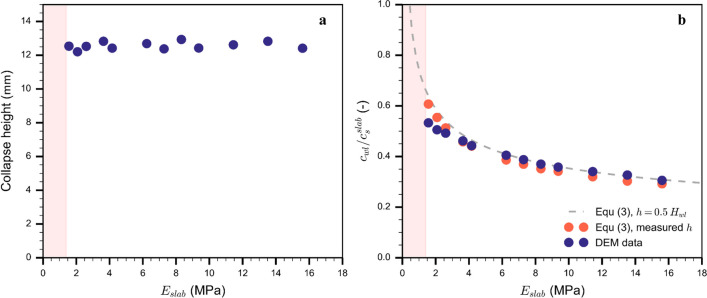
(a) Collapse height as function of slab elastic modulus: DEM data shown by full circles. (b) Normalized steady-state crack speed as function of slab elastic modulus: DEM data shown by dark blue full circles. The dashed grey line shows the semi-empirical model $c=\sqrt[4]{-\frac{g}{2h}\frac{D({E}_{slab})}{\rho H}}+\sqrt[4]{{{\beta }_{1} E}_{wl}}+{\beta }_{2} {\sigma }_{wl}^{th}+\varepsilon$ assuming a collapse height of 50% of the weak layer thickness (1 cm). Orange circles shows speed obtained with the semi-empirical model with the collapse height as obtained in the DEM simulation and shown in (a)

**Fig. 12:**
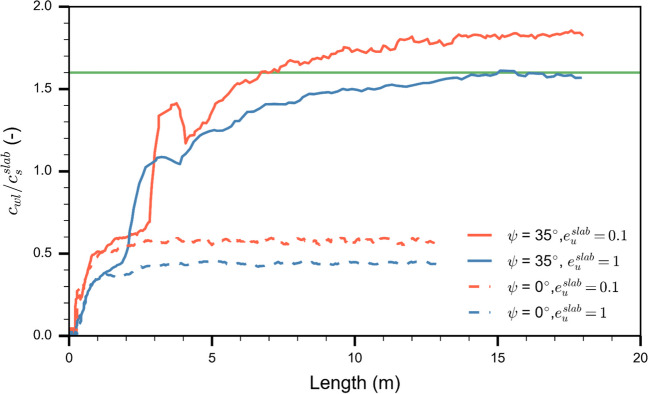
Normalized crack speed along the PST column for different slope angles ($\psi$). The dashed line shows the crack speed evolution for a PST in flat terrain ($\psi$= 0°) and the solid line for a tilted PST ($\psi$= 35°). Orange lines correspond to a restitution coefficient equal to 0.1, i.e., weak layer and slab particles are damped. Blue lines correspond to a slab restitution coefficient equal to 1.0, i.e. no damping applied for slab particles, $e_u=0.1$ for weak layer particles)

**Fig. 13:**
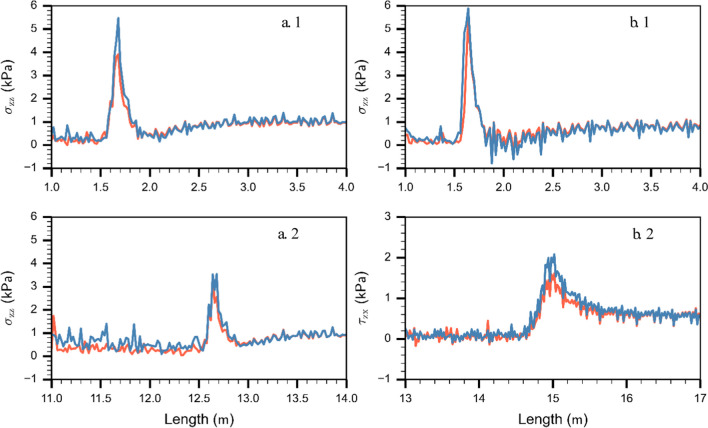
Orange lines correspond to simulation results obtained with slab particle damping and blue lines without damping in the slab, restitution coefficient ${e}_{u}$ equal to 0.1 and 1, respectively. (a.1, a.2) Normal stresses for different crack tip locations (1.7 m and 12.7 m) on flat terrain ($\psi$= 0°). (b1, b2) Normal and shear stresses for different crack tip locations (1.7 m and 15 m) on titled terrain ($\psi$= 35°)

## Appendix 1 Crack speed as function of weak layer parameters for a stiff slab

Figure 10

## Appendix 2 Influence of collapse height on crack speed

Figure 11

## Appendix 3 Influence of damping on crack propagation speed

Figure 12

## Appendix 4 Influence of damping on stresses during crack propagation

Figure 13
